# Supplementary material for: Differentiation of Cytopathic Effects (CPE) induced by influenza virus infection using deep Convolutional Neural Networks (CNN)
Source: PLoS Comput Biol. 2020 May 13;16(5):e1007883. doi: 10.1371/journal.pcbi.1007883 (PMC7279608; doi:10.1371/journal.pcbi.1007883)
Supplement: S1 Table — (DOC) [file pcbi.1007883.s001.doc]

Supporting Information

**S1 Table. Information Table for the Training 1 Initial Testing Data Set**

|  | **Infection condition** | | **Sample numbers** |
| --- | --- | --- | --- |
|  | **Dose (M.O.I.)** | **Time (hpi)** |  |
| **Negative samples** | - | 25 | 100 |
| **Positive samples** | 0.05 | 25 | 150 |
|  | 0.5 | 25 | 150 |

Abbreviation: M.O.I. (multiplicity of infection); hpi (hours post infection)
