# Supplementary material for: Differentiation of Cytopathic Effects (CPE) induced by influenza virus infection using deep Convolutional Neural Networks (CNN)
Source: PLoS Comput Biol. 2020 May 13;16(5):e1007883. doi: 10.1371/journal.pcbi.1007883 (PMC7279608; doi:10.1371/journal.pcbi.1007883)
Supplement: S2 Table — (DOC) [file pcbi.1007883.s002.doc]

Supporting Information

**S2 Table. Transfer Learning Model Comparison of Training 1 and Training 2**

|  | | **Training 1** | **Training 2** |
| --- | --- | --- | --- |
| **Training numbers** | | 601 | 503 |
| **Training accuracy** | | 0.7454 | 0.7654 |
| **Testing numbers** | | 400 | 498 |
| **Testing accuracy** | | 0.7575 | 0.7650 |
| **Accuracy of additional testing data** | 16 hpi Pos+Neg^a^ (415)* | 0.6698 | 0.6626 |
|  | 16 hpi Neg (140) | 0.0213 | 0 |
|  | 16 hpi Pos (275) | 1 | 1 |
|  | 16 hpi 0.5M.O.I. (162) | 1 | 1 |
|  | 16 hpi 0.05M.O.I. (113) | 1 | 1 |
|  | 28 hpi Pos+Neg ^a^ (400) | 0.7 | 0.6925 |
|  | 28 hpi Neg (123) | 0.0243 | 0 |
|  | 28 hpi Pos (277) | 1 | 1 |
|  | 40 hpi Pos+Neg (375) | 0.7333 | 0.7253 |
|  | 40 hpi Neg (103) | 0.0291 | 0 |
|  | 40 hpi Pos (272) | 1 | 1 |
| **Accuracy of other viruses data** | HSV-1 | 0.15 | 0 |
|  | HSV-2 | 0 | 0 |
|  | RSV | 0 | 0 |
|  | Parainfluenza virus | 0 | 0 |
|  | Coxsackievirus B3 | 0 | 0 |
|  | Adenovirus | 0 | 0 |
| ^a^ Pos+Neg: positive samples and negative samples | | | |
| * Numbers in the brackets represent the amount of photos | | | |
| Abbreviation: Pos, positive samples; Neg, negative samples; PPV, positive predictive value; NPV, negative predictive value; HSV-1, herpes simplex virus type 1; HSV-2, herpes simplex virus type 2; RSV, respiratory syncytium virus. | | | |
|  |  |  |  |
