# Supplementary material for: Differentiation of Cytopathic Effects (CPE) induced by influenza virus infection using deep Convolutional Neural Networks (CNN)
Source: PLoS Comput Biol. 2020 May 13;16(5):e1007883. doi: 10.1371/journal.pcbi.1007883 (PMC7279608; doi:10.1371/journal.pcbi.1007883)
Supplement: S3 Table — (DOC) [file pcbi.1007883.s003.doc]

Supporting Information

S3 Table. Comparison of Training 1 and Training 2 with 1200 epochs weights on other viruses infected images at earlier time point

|  | | **Training 1(1200 epochs)** | | | **Training 2(1200 epochs)** | | |
| --- | --- | --- | --- | --- | --- | --- | --- |
| **stain** | | 16hpi | 25hpi | 28hpi | 16hpi | 25hpi | 28hpi |
| **Accuracy of other viruses data** | HSV-1 | 1 | 1 | 1 | 1 | 1 | 1 |
|  | HSV-2 | 1 | 1 | 1 | 1 | 1 | 1 |
|  | RSV | 1 | 1 | 1 | 1 | 1 | 1 |
|  | Parainfluenza virus | 1 | 1 | 1 | 1 | 1 | 1 |
|  | Coxsackievirus B3 | 1 | 1 | 1 | 1 | 1 | 1 |
|  | Adenovirus | 1 | 1 | 1 | 1 | 1 | 1 |
| Abbreviation: HSV-1, herpes simplex virus type 1; HSV-2, herpes simplex virus type 2; RSV, respiratory syncytium virus | | | | | | | |
