# Supplementary material for: Differentiation of Cytopathic Effects (CPE) induced by influenza virus infection using deep Convolutional Neural Networks (CNN)
Source: PLoS Comput Biol. 2020 May 13;16(5):e1007883. doi: 10.1371/journal.pcbi.1007883 (PMC7279608; doi:10.1371/journal.pcbi.1007883)
Supplement: S4 Table — (DOC) [file pcbi.1007883.s004.doc]

Supporting Information

S4 Table. Comparison of Training 1 and Training 2 with 1200 epochs weights on other strains of influenza virus data

|  | | **Training 1 (1200 epochs)** | | | **Training 2 (1200 epochs)** | | |
| --- | --- | --- | --- | --- | --- | --- | --- |
| **strain** | | Pandemic H1N1/09 | H3N2 | flu B | Pandemic H1N1/09 | H3N2 | flu B |
| **Accuracy of additional testing data** | 16 hpi Pos | 0.1869*** | 0.9308*** | 0.9796 | 0.2886** | 0.9471*** | 0.9634*** |
|  | 16 hpi 0.5M.O.I. | 0.3553*** | 0.9090 | 1 | 0.4958** | 0.9090*** | 1*** |
|  | 16 hpi 0.05M.O.I. | 0.0240*** | 0.9520*** | 0.9593 | 0.0879 | 0.9840*** | 0.9268*** |
|  | 25hpi Pos | 0.98* | 0.9767* | 0.9795* | 1** | 0.9933** | 0.9713 |
|  | 28 hpi Pos | 0.9835 | 1*** | 0.9180 | 0.9917*** | 0.9876** | 0.8360** |
|  | 40 hpi Pos | 0.8724 | 0.8215 | 0.9224 | 0.7325*** | 0.6639*** | 0.8816*** |
| Abbreviation: M.O.I. (multiplicity of infection); hpi (hours post infection); Pos, positive samples. | | | | | | | |
