# Supplementary material for: Differentiation of Cytopathic Effects (CPE) induced by influenza virus infection using deep Convolutional Neural Networks (CNN)
Source: PLoS Comput Biol. 2020 May 13;16(5):e1007883. doi: 10.1371/journal.pcbi.1007883 (PMC7279608; doi:10.1371/journal.pcbi.1007883)
Supplement: S5 Table — (DOC) [file pcbi.1007883.s005.doc]

Supporting Information

S5 Table. CNN Model with quarter size images

|  | **Accuracy** |
| --- | --- |
| **Training data** | 0.7755 |
| **Testing data** | 0.75 |
