# Supplementary material for: Differentiation of Cytopathic Effects (CPE) induced by influenza virus infection using deep Convolutional Neural Networks (CNN)
Source: PLoS Comput Biol. 2020 May 13;16(5):e1007883. doi: 10.1371/journal.pcbi.1007883 (PMC7279608; doi:10.1371/journal.pcbi.1007883)
Supplement: Attachment — Submitted filename: Reviewer_Plos_com v2.docx [file pcbi.1007883.s006.docx]

Dear Editor,

We would like to thank you and the reviewers for giving us the opportunity to improve our manuscript entitled “Differentiation of Cytopathic Effects (CPE) Induced by Influenza Virus Infection Using Deep Convolutional Neural Networks (CNN)”. Our point-to-point response to the reviewers' comments and queries are listed below.

Reviewer #1: The authors Chang et al. described a CNN that classifies samples into two categories (infected with flu vs not infected with flu) based on images of cells cultured with sample after various times. Two training/validation sets have been constructed and results were similar. The results appear to be robust and have clinical application.

Major issues:
The training data set (images, and their labels) are not provided. It would be better if the code and trained model is also available.

**Reply**: Thank you for your comment. We have uploaded our training data set, testing data set, experiment data set, the code, and the trained model to the website (Github)(

<https://github.com/b96404011/influenzaCPE_CNN).(Line>s 456-457)

.

When images of cells infected with other viruses are used as the input, what is the output?

**Reply:** Thank you for your comment. We used the code “model.predict_classes” to examine the images of MDCK cells infected by other viruses. Before we examined it, we didn’t know the specificity of the model. When images of cells infected with other viruses were used as the input, the output was negative as we have expected.

The data in Table 4 indicated that for RSV, training 2, 1200 epochs, accuracy is 1 but the best one is 0.35. What does the column "best one" indicate? Why is the best one is 0.35 and 1200 epoch is 1? Should the best one be better than 1200 epochs (most of the rows are but not always)?

**Reply**: Thank you for your comment. In the result section “Result of other weights”, we mentioned that we tested the weights saved in training, and the “best one” means the best result in those saved weights. We edited the term to “saved weights” to avoid the misconception. Those saved weights were saved within 100 epoch. However, these results were not as good as the final weights after 1200 epoch training. It probably implies that the model is convergent quickly in the beginning, but 1200 epochs were necessary.

Based on the images provide for the other virus-infected cells, some of them have apparent changes in cell morphology. If they are not classified as influenza damage, are they classified as no damage? (because the CNN only has two outputs).

**Reply**: Thank you for your comment. Considering the model was trained to promote the recognition of influenza-infected cells, those images were classified as no damage.

The manuscript can use some better organization regarding the introduction of training1 vs. training2. Training 2 data appeared before they were introduced, I suspect this was a response to a prior review of some sort.

**Reply:** Thank you for your comment. The only reason we put the training 2 data with training 1 data in Table 2 was because we thought it will help the readers to compare the results. If the reviewer prefers, we can extract the training 2 data from Table 2 and create a new Table 6.

Minor issues

The manuscript could use some editing of the English Language.

For example:

Page 10: line 145: were differentiated correctly: Do the author mean identified?

**Reply**: Thank you for your comment. The sentence has been revised as “Moreover, all of the 300 positive pictures were identified correctly, with no error, resulting in a 100% negative predictive value.”.

Page 19: line 220: resigned training data? What is "resigned" referring to?

**Reply**: Thank you for your comment. It should be “reassigned training data”.

Reviewer #2: The manuscript by Want et al. describes a deep convolutional neural network (CNN) approach to detect influenza induced cytopathic effects from microscopic images of influenza infected MDCK cells. The aim is to develop a fast and accurate tool for rapid influenza diagnoses in clinics. Deep learning approaches have been proven to be extremely powerful for identifying objects of interests from images in the past decade. Thus, the approach to the problem was well chosen in the study. The authors showed that trained model can achieve a very high percentage of correct recognition (99.75%). Although the results look convincing, my enthusiasm about the study is dampened by a couple of major concerns and some minor concerns.

First of all, the images used to train the model are images of cells infected by a single virus strain (PR8). While the authors tested the model against a variety of other non-influenza viruses, I think it is critical/essential to test how the model works with other influenza strains. This is because we usually do not know the virus strain that a person is infected with as a priori. The model is useful only when it can correctly predict the presence of influenza virus irrespective of the strain a cell is infected with.
**Reply**: Thank you for your comment. The PR8 virus strain is a laboratory reference strain for influenza study and the CPE induced by PR8 is representative to most influenza viruses. That is why we used PR8 to examine the patterns of CPE in infected MDCK cells. However, we have collected additional images infected by other influenza virus strains to support this. About 120 images of MDCK cells infected by pandemic H1N1/09, H3N2, influenza B at 16, 25, 28, and 40 hpi were collected, respectively. We tested these images with our model and the results were tabulated in S4 Table. As expectation, our model can recognize most of them as good as testing data and experiment data, even better. The slight difference might be contributed by different growth kinetics of various influenza strains. A description about the additional test has been added in the revised manuscript.

Line 331-337

“	In this study, we used the PR8 virus strain to conduct the experiment. The PR8 virus strain is a laboratory reference strain for influenza study and the CPE induced by PR8 is representative to most influenza viruses. To examine that our model can recognize influenza CPE induced by different influenza virus strains, we collected about 120 image of MDCK cells infected by pandemic H1N1/09, H3N2, and influenza B at 16, 25, 28, and 40 hpi, respectively. Most results of recognition were as good as our testing data and experiment data, even better (S4 Table). The slight difference might be contributed by the different growth kinetics of influenza strains.”

Second, it is not clear to me how this method is compared with other method in terms of accuracy and time needed for diagnoses. Although the authors discussed and compared the performance of their model with the performance of R-mix, the comparison does not seem to be fair, because the tests were done using different sets of data and images collected from different experiment with different protocols. Furthermore, the CNN model may be an automation method and a useful alternative to visual examination, for example by a medical technologist. However, it is not clear to me how much (quantitative) benefit one gains as compared to visual examination, e.g. how much more accurate the CNN model performs compared to visual examination and how much time it saves. Overall, the advantages and significance of this approach in terms of clinical use is not clearly discussed and highlighted. This really limit the enthusiasm about the proposed approach.

**Reply**: Thank you for your comment. We added comparison between Training 1 , Training 2 and visual examination in the revised manuscript (Table 5). A better performance of our model as compared to the visual examination was shown in Table 5. A description about the comparison has been added in the revised manuscript.

Line 265-269

“To examine our model can reduce the time of identifying influenza-infected MDCK cells, we compared our model with visual examination. As shown in Table 5, both Training 1 and Training 2 performed significantly higher accuracy than visual examination. The significantly higher value of negative predictive value of Training 1 and Training 2 implicated that our model can recognize influenza-induced CPE images at earlier time points as compared to visual examination.

**Table 5. Comparison of Training 1 , Training 2 and visual examination**

|  | **Accuracy** | **PPV** ^a^ | **NPV** ^b^ | **confusion matrix** | | |
| --- | --- | --- | --- | --- | --- | --- |
|  |  |  |  | **Actual** | **Predicted** | |
|  |  |  |  |  | **Pos** | **Neg** |
| **Training 1** | 0.9975^***^ | 0.9966^*^ | 1^***^ | **Pos** | 300 | 0 |
|  |  |  |  | **Neg** | 1 | 99 |
| **Training 2** | 0.9457^***^ | 0.9673 | 0.8846^***^ | **Pos** | 356 | 15 |
|  |  |  |  | **Neg** | 12 | 115 |
| **visual examination** | 0.8025 | 1^*^ | 0.5586 | **Pos** | 221 | 79 |
|  |  |  |  | **Neg** | 0 | 100 |
| Abbreviation: Pos, positive samples; Neg, negative samples; PPV, positive predictive value; NPV, negative predictive value.  ^a^ PPV=numbers of true positives/(numbers of true positives+ numbers of false positives). The ideal value of the PPV for a perfect test will be 1, while the worst value will be zero.  ^b^ NPV=numbers of true negatives/(numbers of true negatives+ numbers of false negatives). The ideal value of the NPV for a perfect test will be 1, while the worst value will be zero  ^***^: p-value <0.001 | | | | | | |
| ^*^: p-value <0.05 | | | | | | |

”

Third, in Fig 4, the authors used the model to test against non-influenza viruses and concluded that ’no cytopathic effect was observed in MDCK cells infected by other viruses’. It is not clear to me what analysis was exactly performed. Did the author use the model trained on influenza infected and mock MDCK cells to classify images of cells infected by non-influenza viruses; or the authors retrained the model including the images of cells infected by non-influenza viruses? In the images with other virus infections, is that indeed ‘there is no cytopathic effects’; or other viruses cause cytopathic effects that are different from influenza? I also notice that the images were all taken at 40phi (for other viruses). What about other time points?
**Reply**: Thank you for your comment. We use the model trained on influenza infected and mock MDCK cells to classify images of cells infected by non-influenza viruses. We did not retrain the model by including the images of cells infected by non-influenza viruses. In the clinical laboratory, MDCK cells are mainly used to isolate influenza viruses. Some viruses, such as parainfluenza viruses, RSV and HSV, have been reported to cause CPE in MDCK cells, while others can not replicate in MDCK cells. For those viruses which can not replicate in MDCK cells, no cytopathic effects can be observed. In our study results, all of the images from non-influenza virus infected cells were not recognized as influenza-infected cells. The reasons for the images being taken at 40phi (for other viruses) is that CPE development is a continuous process and the last time point of influenza-infected images was 40 hpi. We assumed that if the training model can not recognize significant cell morphology changes at 40 hours as influenza-infected CPE, it should not be able to recognize any changes before 40 hours. However, to confirm this assumption, we added a supplementary Table 3, which included data from 20 images of DMCK cell infected by these six viruses at 16, 25, and 28 hpi, respectively. The results were consistent with the 40 hpi images. A description about the additional test has been added in the revised manuscript.

Line 175-177:

“In addition, twenty images were taken for each virus infected cells at 16, 25, and 28 hpi and validated by the model. The results were consistent with those using the 40 hpi images (S3 table).”

Some minor points below:
Lines 186- 201, it is not clear to me how the accuracy of prediction of images taken at different time points were done. Did the authors retrain the model; or just used the model trained on the Training dataset described in the earlier text?

**Reply**: Thank you for your comment. We used the model trained on the Training dataset described in the earlier text. To make it clearer, we revised the sentence as

“We fed more experiment data taken at earlier time points, 16 hpi, into our trained model (Fig 3D-F).”.(Line 190)

Line 194-195, Can the authors test why the accuracy drops at 40hpi? It is not sufficient to speculate the cause.

**Reply**: Thank you for your comment. A higher percentages of CPE developed at 40 hours after influenza infection and in some areas, infected cells detached from the plates, which resulted in empty background in the 40 hpi images of influenza-infected MDCK cells. A more thorough discussion about this observation was added in Lines 323-330.

Line 198, typo ‘act’ -> ‘fact’?

**Reply**: Thank you for your comment. It should be “fact”
