# Supplementary material for: Differentiation of Cytopathic Effects (CPE) induced by influenza virus infection using deep Convolutional Neural Networks (CNN)
Source: PLoS Comput Biol. 2020 May 13;16(5):e1007883. doi: 10.1371/journal.pcbi.1007883 (PMC7279608; doi:10.1371/journal.pcbi.1007883)
Supplement: Attachment — Submitted filename: Reviewer_Plos_com R2.docx [file pcbi.1007883.s007.docx]

Dear Editor,

We would like to thank you and the reviewers for giving us the opportunity to improve our manuscript entitled “Differentiation of Cytopathic Effects (CPE) Induced by Influenza Virus Infection Using Deep Convolutional Neural Networks (CNN)”. Our point-to-point response to the reviewers' comments and queries are listed below.

Reviewer #1: The authors need to incorporate the reply to reviewer's question in the revised manuscript. Most of the answers to Reviewer 1 were not reflected in the revision. The authors stated that they changed "best one" to "saved weights". But in table 2, they still used "best one". Also although the code is provided in github (much appreciated), the data is provided through a link. The link time out when I tried to download the data.
**Reply:** We have incorporated most of the changes to the revised manuscript except creating a new Table 6 (response to Q5). However, if the reviewer prefers to extract the training 2 data from Table 2 and create a new Table 6, we can do that accordingly.

(1) Major issues:
    The training data set (images, and their labels) are not provided. It would be better if the code and trained model is also available.

**Reply**: Thank you for your comment. We have uploaded our training data set, testing data set, experiment data set, the code, and the trained model to the website (Github)(

[https://github.com/b96404011/influenzaCPE_CNN) (Line](https://github.com/b96404011/influenzaCPE_CNN)%20(Line)s 459-460). Because the original merged dataset was huge and it would take 30 minutes to one hour to download, we divided the data and uploaded them individually in the Github. Original merged input data can still be downloaded from the linkage ([dataset download](https://ppt.cc/f8uK5x)).

(2) When images of cells infected with other viruses are used as the input, what is the output?

**Reply:** Thank you for your comment. We used the code “model.predict_classes” to examine the images of MDCK cells infected by other viruses. Before we examined it, we didn’t know the specificity of the model. When images of cells infected with other viruses were used as the input, the output was negative as we have expected. The reply has been incorporated in Line 434.

(3) The data in Table 4 indicated that for RSV, training 2, 1200 epochs, accuracy is 1 but the best one is 0.35. What does the column "best one" indicate? Why is the best one is 0.35 and 1200 epoch is 1? Should the best one be better than 1200 epochs (most of the rows are but not always)?

**Reply**: Thank you for your comment. In the result section “Result of other weights”, we mentioned that we tested the weights saved in training, and the “best one” means the best result in those saved weights. We edited the term to “saved weights” to avoid the misconception. Those saved weights were saved within 100 epoch. However, these results were not as good as the final weights after 1200 epoch training. It probably implied that the model was convergent quickly in the beginning, but 1200 epochs were necessary. The reply has been incorporated in Lines 279-281.

(4) Based on the images provide for the other virus-infected cells, some of them have apparent changes in cell morphology. If they are not classified as influenza damage, are they classified as no damage? (because the CNN only has two outputs).

**Reply**: Thank you for your comment. Considering the model was trained to promote the recognition of influenza-infected cells, those images were classified as no damage. (Line 434)

(5) The manuscript can use some better organization regarding the introduction of training1 vs. training2. Training 2 data appeared before they were introduced, I suspect this was a response to a prior review of some sort.

**Reply:** Thank you for your comment. The only reason we put the training 2 data with training 1 data in Table 2 was because we thought it will help the readers to compare the results. If the reviewer prefers, we can extract the training 2 data from Table 2 and create a new Table 6.

(6) Minor issues

The manuscript could use some editing of the English Language.

For example:

Page 10: line 145: were differentiated correctly: Do the author mean identified?

**Reply**: Thank you for your comment. The sentence has been revised as “Moreover, all of the 300 positive pictures were identified correctly, with no error, resulting in a 100% negative predictive value.”. (Line 145 in the revised manuscript)

Page 19: line 220: resigned training data? What is "resigned" referring to?

**Reply**: Thank you for your comment. It should be “reassigned training data”(Line 222 in the revised manuscript).

Reviewer #2: All my concerns are appropriately addressed. I appreciate the author's efforts to collected additional data (images) of cells infected by other strains. This added a lot of robustness to the model.

**Reply**: Thank you.
